# ROS-generating, pH-responsive and highly tunable reduced graphene oxide-embedded microbeads showing intrinsic anticancer properties and multi-drug co-delivery capacity for combination cancer therapy

**DOI:** 10.1080/10717544.2022.2100512

**Published:** 2022-07-31

**Authors:** Adilakshmi Boddu, Sreekanth Reddy Obireddy, Dahong Zhang, K. S. V. Krishna Rao, Wing-Fu Lai

**Affiliations:** a Polymer Biomaterial Design and Synthesis Laboratory, Department of Chemistry, Yogi Vemana University, Kadapa, India; b Department of Chemistry, Sri Krishnadeveraya University, Anantapuramu, India; c Department of Urology, Zhejiang Provincial People’s Hospital, Hangzhou Medical College, Zhejiang, China; d Department of Applied Biology and Chemical Technology, Hong Kong Polytechnic University, Hong Kong, China

**Keywords:** Carbohydrate, curcumin, reduced graphene oxide, anti-cancer, chitosan, sodium alginate

## Abstract

The development of effective carriers enabling combination cancer therapy is of practical importance due to its potential to enhance the effectiveness of cancer treatment. However, most of the reported carriers are monofunctional in nature. The carriers that can be applied to concomitantly mediate multiple treatment modalities are highly deficient. This study fills this gap by reporting the design and fabrication of ROS-generating carbohydrate-based pH-responsive beads with intrinsic anticancer therapy and multidrug co-delivery capacity for combination cancer therapy. Sodium alginate (SA) microspheres and reduced graphene oxide (rGO)-embedded chitosan (CS) beads are developed via emulsion-templated ionic gelation for a combination therapy involving co-delivery of curcumin (CUR) and 5-fluororacil (5-FU). Drug-encapsulated microbeads are characterized by FTIR, DSC, TGA, XRD, and SEM. 5-FU and CUR-encapsulated microbeads are subjected to *in vitro* drug release studies at pH 6.8 and 1.2 at 37 °C. Various release kinetic parameters are evaluated. The results show that the Korsmeyer-Peppas model and non-Fickian release kinetics are best suited. The microspheres and microbeads are found to effectively act against MCF7 cells and show intrinsic anticancer capacity. These results indicate the promising performance of our beads in mediating combination drug therapy to improve the effectiveness of cancer treatment.

## Graphical Abstract

**Figure:**
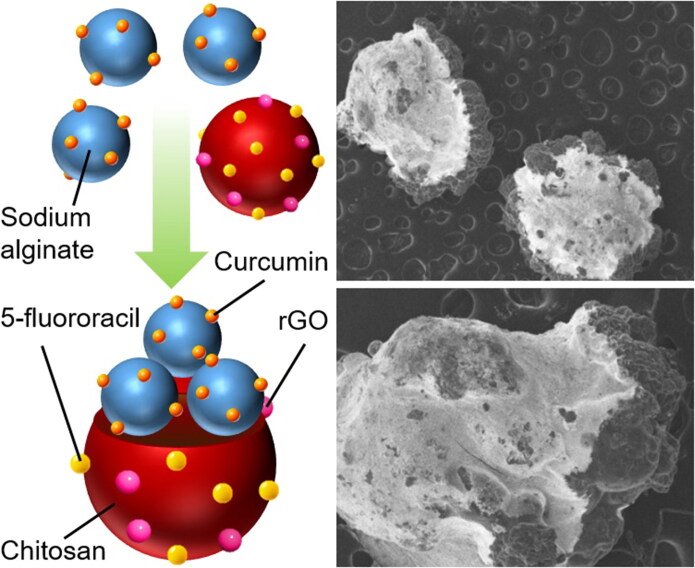

## 1. Introduction

Polymeric matrices for targeted drug delivery have attracted extensive interest over the years (Steichen et al., 2013; Lai et al., 2020; Lai, Deng, et al. 2021; Lai, Huang, et al. 2021). Carbohydrate polymers allow for the development of different types of pharmaceutical dosage formulations, such as oral, parenteral, semisolid, and controlled/sustained drug delivery systems (Lai et al., 2019, 2020; Ding et al., 2021; Lai, 2022). These dosage formulations release different types of drugs (i.e., hydrophilic and hydrophobic) in a synchronized manner and enable constant release of formulations over extended periods (Hughes, 2005). In addition, polymeric drug carriers control the drug release rate, and may increase the safety, efficacy, and bioavailability of the drugs (Allen & Cullis, 2013). Sodium alginate (SA) is an anionic polysaccharide obtained from marine brown algae (Pawar & Edgar, 2012; Szabo et al., 2020). It has been used in food and pharmaceutical applications because it cannot only form gels in mild conditions but is also biocompatible, hydrophilic, and easy to work with (Obireddy et al., 2020). In the last few decades, it has been used in controlled, sustained, and targeted drug delivery (Pandey et al., 2019; Pan et al., 2021). Apart from polymeric materials, graphene-based materials such as graphite, graphene oxide (GO), and reduced graphene oxide (rGO) have been studied for various applications ranging from diagnostics to drug delivery owing to their unique physical and chemical properties and, renewability. Because it has epoxide, hydroxyl, and carboxyl groups in its structure, oxygen-rich GO has a variety of applications (Joshi et al., 2020). GO has a large surface area. The oxygen-containing groups of graphene derivatives provide high drug loading efficiency, good dispersion, and ease of functionalization. On the other hand, rGO has a planar structure, which makes it very good at loading drugs and strong at absorbing near-infrared light (Zhou et al., 2009; Kim et al., 2013).

Despite the advances in carrier development, carriers that has been practically and effectively used in cancer therapy have been scant till now (Lai & Lin, 2015; Li et al., 2021). In fact, right now cancer is still a major cause of mortality and morbidity throughout the world (Ma & Yu, 2006). Conventional chemotherapy is ineffective in eradicating cancer cells, and can lead to drug resistance, which happens because of two important reasons: (i) the failure to deliver drugs to the tumor site; and (ii) specific genetic alternations in cancer cells (Gottesman, 2002). Over the years, a large variety of carriers have been developed and reported in the literature; however, most of them are designed to mediate single-drug delivery (Reddy et al., 2020; Sreekanth Reddy et al., 2021). Owing to the development of drug resistance among cancer cells, using a single agent is often ineffective (Gottesman, 2002; Noguchi et al., 2009; Yang et al., 2021). An effective strategy to combat cancer, therefore, requires a combination approach. Combination therapies, which refers to either the simultaneous administration of two or more pharmacologically active agents or the combination of different types of therapy (Broxterman & Georgopapadakou, 2005; Greco & Vicent, 2009), can minimize the side effects and improve prognosis, thereby showing the potential to treat diverse tumors and infectious diseases (Woodcock et al., 2011). Carriers that can be used to deliver more than one bioactive agent at the same time are very useful because they make multi-drug therapy possible (Obireddy and Lai 2021a). Recently, a study made multi-component hydrogel beads with rGO that could be used to deliver multiple bioactive agents (Obireddy and Lai 2021a). In another study, Reddy and his team (Obireddy and Lai 2021b) came up with hydroxyethyl starch microparticles that could be used to deliver both ketoprofen and ofloxacin at the same time. In the present study, we have synthesized microparticles embedded in microbeads to co-deliver doxorubicin and 5-fluorouracil for cancer treatment (Obireddy and Lai 2021a). A delivery system loaded with two or more anticancer drugs is expected to have specific activity on cells at different growth stages and act synergistically. As a result, the combination therapy should be able to get around drug resistance among cancer cells and make each drug more effective. The developed microbeads and rGO were characterized by different techniques such as Fourier-transform infrared (FTIR), spectroscopy, X-ray diffraction (XRD), differential scanning calorimetry (DSC), thermogravimetric analysis (TGA) and scanning electron microscopy (SEM). In addition to this encapsulation efficiency, drug release kinetics, in vitro toxicity and ROS generating capacity of microbeads were also studied.

## 2. Experimental

### 2.1. Materials and methods

Sodium alginate (SA), chitosan (CS), curcumin (CUR), 5-fluorouracil (5-FU), and pentasodium tripolyphosphate (STPP) were purchased from Sigma-Aldrich (St. Louis, MO, USA). Absolute ethanol, calcium chloride, and tween 80 were purchased from Sd. Fine chemicals (Mumbai, India). Millipore water was used throughout the study. rGO was synthesized in accordance with our earlier research (Boddu et al., 2021).

### 2.2. Preparation of microparticles and microbeads

Emulsification followed by the gelation process was used to make curcumin-loaded microspheres. In brief, 50 mg of curcumin was dispersed in 5 mL of ethanol by magnetic stirring for 3 minutes and then transferred to 10 mL of 3% SA solution. The mixture was kept under stirring until the formation of a homogenous solution. The CUR-alginate mixture was emulsified in liquid paraffin oil at a ratio of 1:10 with 2% v/v tween 80 as a surfactant and kept under mechanical stirring at 300 rpm to produce a water/oil emulsion. Then a 10% w/v calcium chloride solution was introduced drop-wise into the emulsion for gelation while keeping the stirring speed constant for 90 minutes. After that, the formed microspheres (SA-CUR) were filtered and washed with petroleum ether before being air-dried at ambient conditions.

**Scheme 1.**
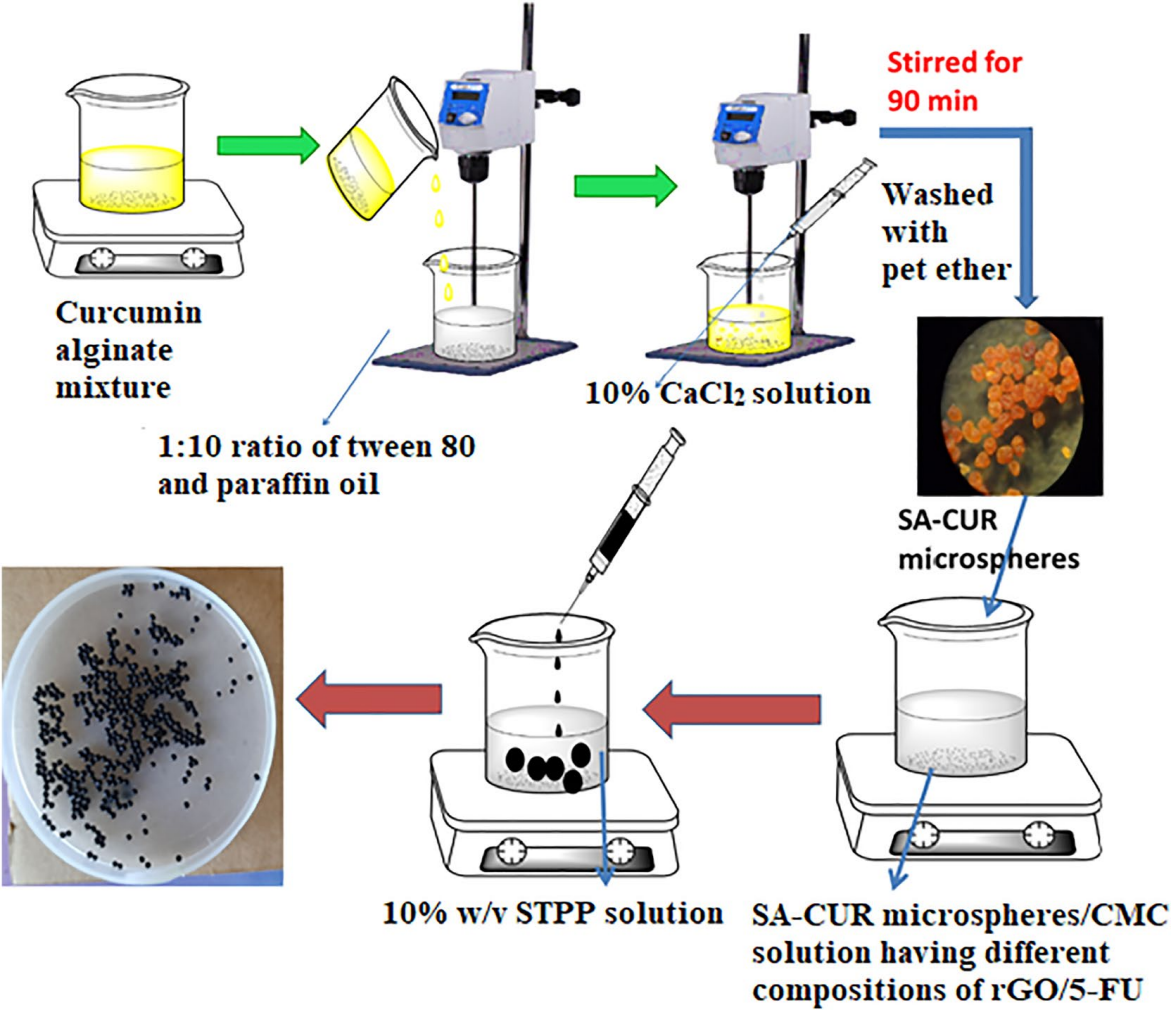
Schematic representation of the procedure used to generate the multi-drug-based delivery system.

To prepare chitosan microbeads, 200 mg of CS was transferred into 10 mL of distilled water containing 1% acetic acid and stirred overnight to get a homogenous solution. 100 mg of 5-FU and 50 mg of rGO were added under stirring up to form a homogeneous solution. Then the mixture was transferred into a 10% w/v STPP solution. The microbeads (CS-rGO-5FU) formed were collected and washed several times with water before being air-dried at ambient conditions. Similarly, SA-5-FU microbeads were synthesized by the above procedure without the addition of rGO. SA microspheres-loaded microbeads (CS-rGO-5FU-SA-CUR) were synthesized by using a similar procedure. During the preparation process, after forming a homogeneous solution containing 5-FU and rGO, the microspheres were added and transferred dropwise into 100 mL of 5% calcium chloride solution to form microbeads. The formed microbeads were collected, filtered, and treated with distilled water. The microbeads were then dried at room temperature and put into airtight containers until they were ready to be used again. Similarly, CS-5FU-SA-CUR microbeads were synthesized by the above procedure without the addition of rGO. The preparation of multi-drug-based delivery system is presented schematically in Scheme 1.

### 2.3. Characterization methods

To find out the interactions between rGO, 5-FU, CUR, SA, CS, and drug-loaded carriers, FTIR spectral analysis was performed in the wavelength range of 400-4000 cm^−1^. To determine the crystalline nature of the samples, XRD analysis (Ultima IV, Rigaku, Japan) was performed and scans were recorded at a scan rate of 10°/min using CuKα radiation. The surface morphology of the developed carriers was characterized by SEM analysis (S4800, Hitachi, Japan). TGA and DSC were used to examine the thermal stability and molecular dispersion of drugs by heating the sample at a rate of 10 °C/min to 600 °C in a nitrogen atmosphere.

### 2.4. In-vitro drug release studies

An eight-basket dissolving analyzer was used to measure the drug release kinetics of drug-loaded carriers (Model: DS 2000, Make: LabIndia, Mumbai, India). In this experiment, 300 mL of PBS (pH 1.2 and 6.8) was used, and 30 mg of microbeads were distributed in PBS using dialysis bags and rotated at a speed of 50 rpm. At regular intervals, 5 mL of dissolution medium was taken out and measured at 288 nm with a UV-vis spectrophotometer. After that, the same volume was filled with new media.

### 2.5. Drug loading content and encapsulation efficiency

Accurately, 10 mg of microbeads were weighed precisely and preserved for 24 hours in 100 mL of PBS (pH 6.8 with 5% absolute alcohol). Afterward, the solution was subjected to sonication and crushed to extract the loaded drugs from the developed drug-loaded samples. A UV-visible spectrophotometer was used to measure the absorbance of the solution at λ_max_=269 and 425 nm for 5-FU and CUR, respectively. The following equations were used to calculate the drug loading content (DLC) and EE.

$$\mathrm{DLC}\;(\%)\;=\frac{\text{Actual drug content}}{\text{Weight of microbeads}/\mathrm{microspheres}}x\;100$$

$$\mathrm{EE}\;(\%)\;=\;\frac{W_{t}}{W_{i}}x\;100$$

where W_t_ is the total amount of 5-FU/CUR in the in drug-loaded carriers and W_i_ is the total amount of 5-FU/CUR initially added during the preparation.

### 2.6. In vitro cytotoxicity and reactive oxygen species analysis

To determine the in vitro cytotoxicity of developed samples, an MMT assay was performed. 200 µL of cell suspension was seeded in a 96-well plate (2 × 10^4^ cells per well) and allowed the cells for 24 h to grow. Then, different concentrations (6.25, 12.5, 25, 50, 100 µg/mL) of test samples (viz., drug-loaded carriers) were added and the plate was incubated for 24 h at 37 °C in a 5% CO_2_ atmosphere. After incubation, the MTT solution (0.5 mg/mL) was added and the plate was incubated for another 3 h. After that, the culture medium was removed and each well was supplemented with 100 μL of dimethyl sulfoxide (DMSO). Later, on a gyratory shaker, the plate was agitated until the absorbance was measured using a UV-vis spectrophotometer at 570 nm. The ROS activity of the developed samples was measured in the same manner as was stated earlier (Obireddy and Lai 2021a).

## 3. Results and discussions

### 3.1. FTIR characterization

**Figure 1.**
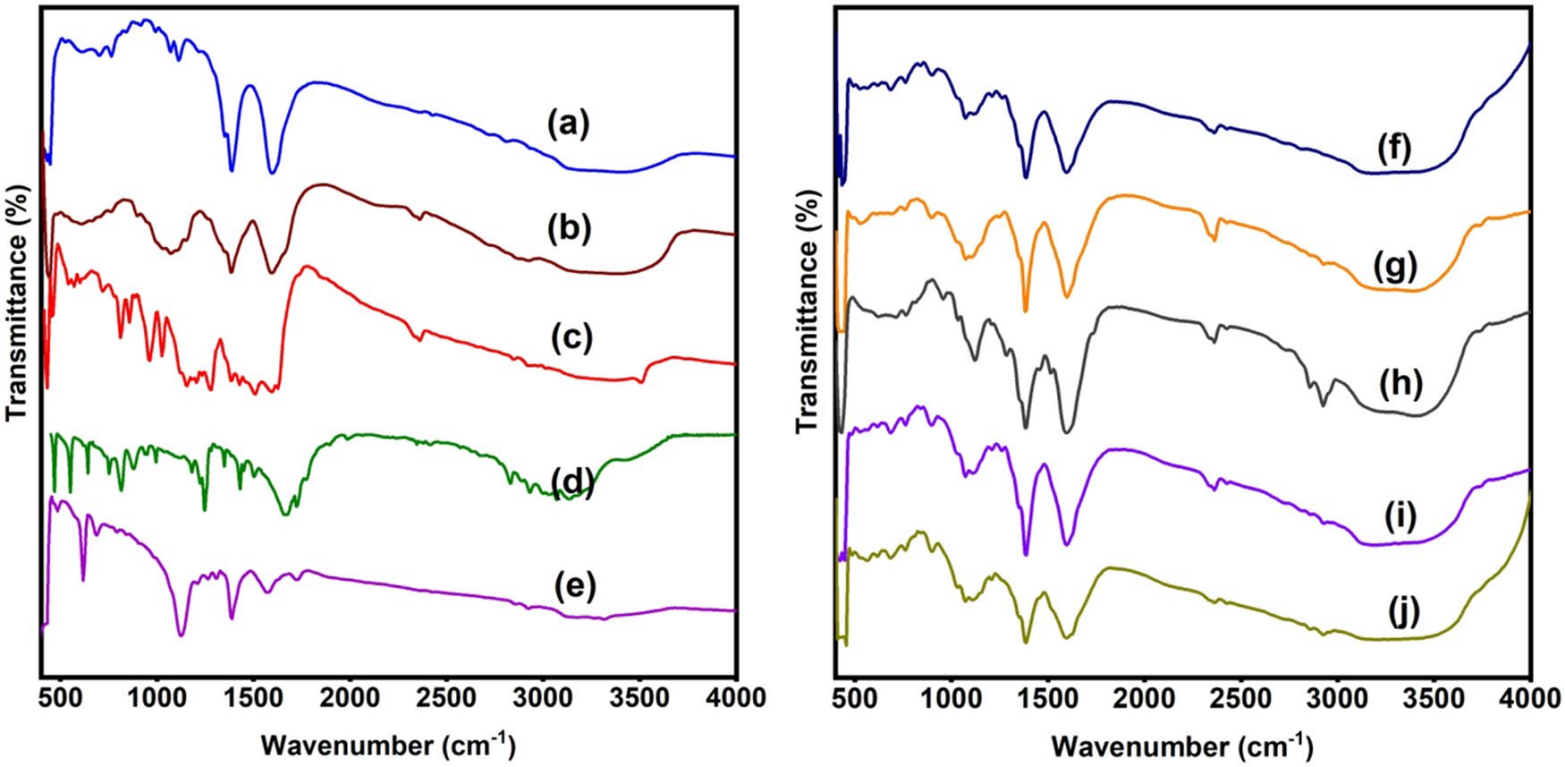
FTIR spectra of a) SA, b) CS, c) CUR, d) 5-FU, e) rGO, f) CS-5FU, g) CS-rGO-5FU, h) SA-CUR, i) CS-5FU-SA-CUR, and j) CS-rGO-5FU-SA-CUR.

To analyze the generation of drug-loaded microspheres and microbeads as well as the interactions between 5-FU, CUR, rGO, and the polymer matrix, FTIR analysis has been performed and the results are displayed in Figure 1. The FTIR spectrum of CUR shows characteristic peaks at 3496 cm^−1^ (O–H stretching frequency), 2923 cm^−1^ (aromatic C–H stretching frequency), 1596 cm^−1^(C = C stretching frequency of the benzene ring skeleton), and 1272 cm^−1^(C–O stretching frequency). The FTIR spectrum of SA shows characteristic peaks at 3406 cm^−1^ (O–H stretching frequency), 1598 and 1388 cm^−1^ (asymmetric C = O stretching frequency). In the case of CUR-loaded SA microspheres, along with the SA peaks, new peaks were observed at 2922 cm^−1^(aromatic C–H stretching frequency) and 1516 cm^−1^(C = C stretching frequency), suggesting that CUR is present in the polymeric matrix (Madhusudana Rao et al., 2015). The FTIR spectrum of CS shows a broad peak at 3419 cm^−1^ (N–H and O–H stretching frequency), 1598 cm^−1^ (N–H bending frequency of amine), 1071 cm^−1^ (C–O stretching frequency), and 610 cm^−1^ (out-of-plane N–H bending frequency) (Varma & Vasudevan, 2020). The FTIR spectrum of rGO shows peaks at 3321 cm^−1^ (O–H stretching frequency), 1723 cm^−1^ (C = O stretching frequency), 1574 cm^−1^(C = C stretching frequency), and 1123 cm^−1^(C–O–C stretching frequency).

The FTIR spectrum of 5-FU shows the peaks at 3009-3139 cm^−1^ (N–H and C–H stretching frequency), 1665 cm^−1^ (C = O stretching frequency), and 1245 cm^−1^(C–F stretching frequency). In the case of 5-FU-loaded microbeads, along with the SA peaks, new peaks were found at 1041 and 762 cm^−1^ (C–F stretching and bending frequency), suggesting that 5-FU is present in the polymeric matrix. After the incorporation of rGO into CS-5FU polymeric matrix, the peak at 1723 cm^−1^ disappears, suggesting that rGO interacts with the polymer matrix and 5-FU. Furthermore, the peak at 1598 cm^−1^ of CS is shifted to the lower side (1596 cm^-1^), which may be due to the interaction between the NH_2_ group of CS and the C = O group of rGO. In addition, the C–F stretching frequencies (1041 and 762 cm^−1^) are found, indicating that 5-FU and rGO are present in the polymer matrix (Piao & Chen, 2016). Similarly, in the case of CS-5FU-SA-CUR and CS-rGO-5FU-SA-CUR microbeads, C–F and C = C stretching frequencies are found, indicating that both 5-FU and CUR are present in the polymeric matrix.

### 3.2. XRD analysis

**Figure 2.**
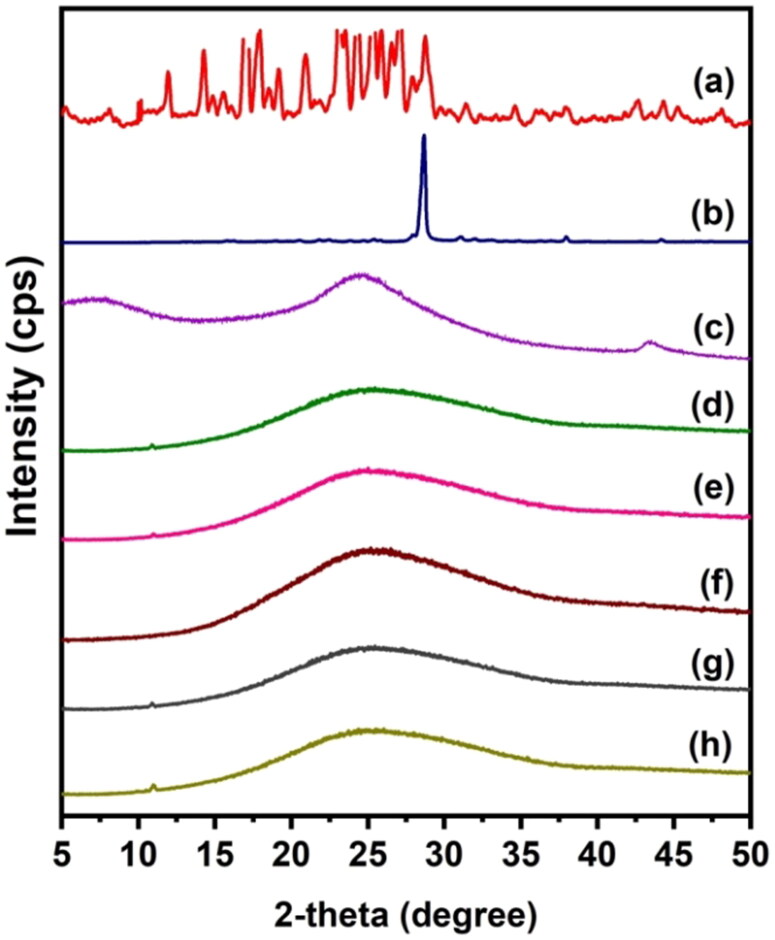
XRD spectra of (a) CUR, (b) 5-FU, (c) rGO, (d) CS-5FU, (e) CS-rGO-5FU, (f) SA-CUR, (g) CS-5FU-SA-CUR, (h) CS-rGO-5FU-SA-CUR.

To find out the dispersion of drugs in the polymeric matrix and the crystalline nature of rGO, 5-FU, and CUR, XRD analysis was performed, and the results are displayed in Figure 2. 5-FU exhibits an XRD pattern with a peak at 28.68^°^, whereas CUR has numerous peaks between 12^°^ and 28^°^. This demonstrates that 5-FU and CUR are crystalline compounds. The absence of these peaks in drug-loaded beads suggests that the crystalline phase of the drug molecules has been converted to an amorphous phase. Moreover, the XRD pattern of rGO reveals a significant peak between 20^o^ and 30^o^. This validates the synthesis of rGO from graphite (Dhanavel et al., 2020) and is in good agreement with that reported by Reddy and Lai, (Obireddy and Lai 2021a) who similarly noticed a prominent peak at around 20–30 in the XRD pattern of rGO. The diffraction peak of rGO, on the other hand, is lost from the XRD patterns of both CS-rGO and CS-rGO-5FU-SA-CUR. This shows that rGO loses its crystalline nature in the polymer matrix and is spread out as nanosheets in the microbeads.

### 3.3. SEM analysis

**Figure 3.**
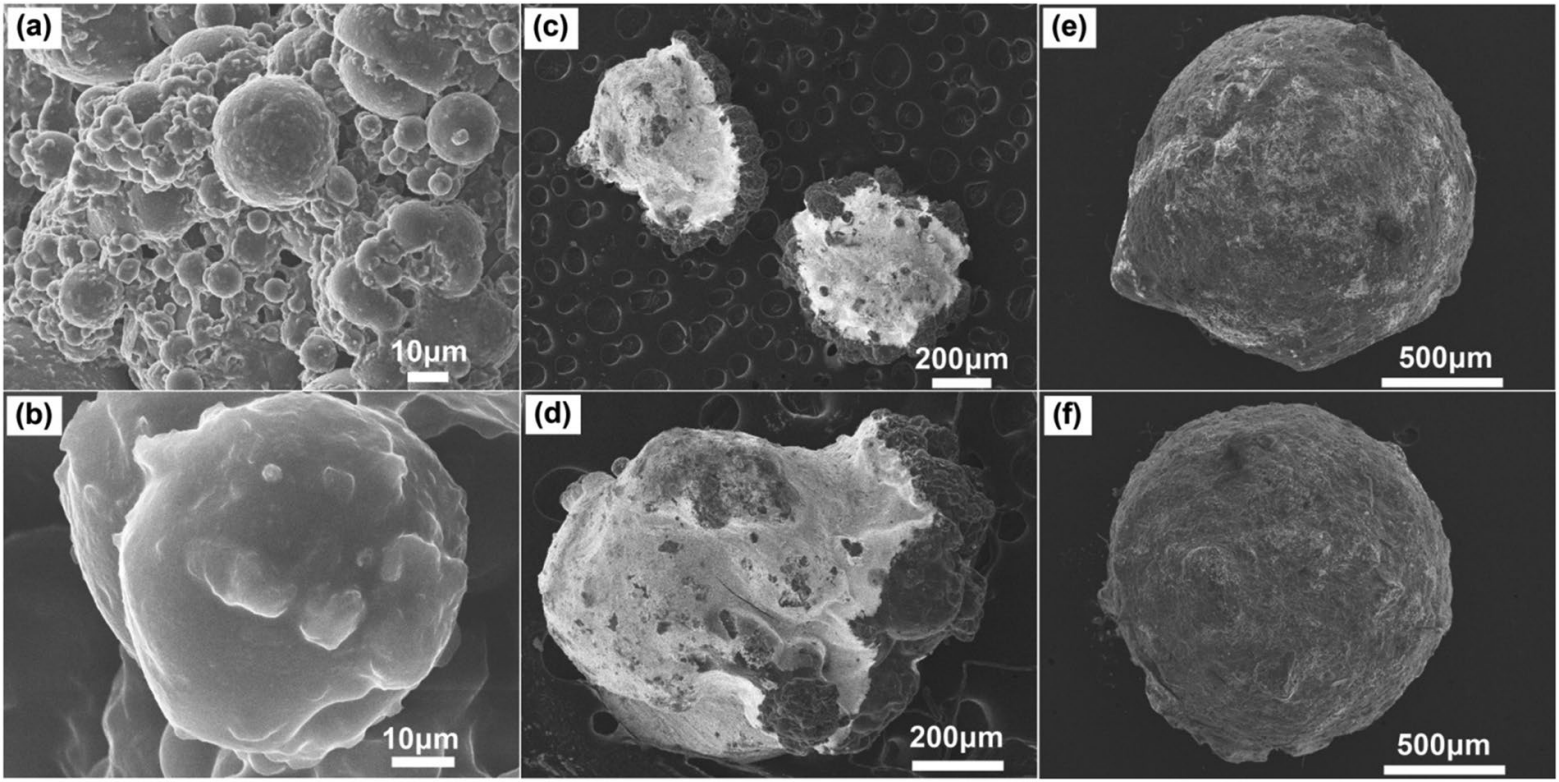
The SEM images of SA-CUR microspheres (a & b), CS-5FU-SA-CUR microbeads (c & d), and CS-rGO-5FU-SA-CUR (e & f).

To examine the external morphological features of the generated microspheres and microbeads, SEM analysis has been done, and the results are shown in Figure 3. It is observed that the microspheres have a spherical form and a rough surface. The typical microsphere diameter is between 30 and 50 μm. After the inclusion of microspheres into microbeads, the outer surface exhibits an increase in roughness, indicating the presence of microspheres within the polymer matrix. SEM analysis determines that the average diameter of the microbeads is between 1200 and 1600 μm.

### 3.4. Thermal analysis

**Figure 4.**
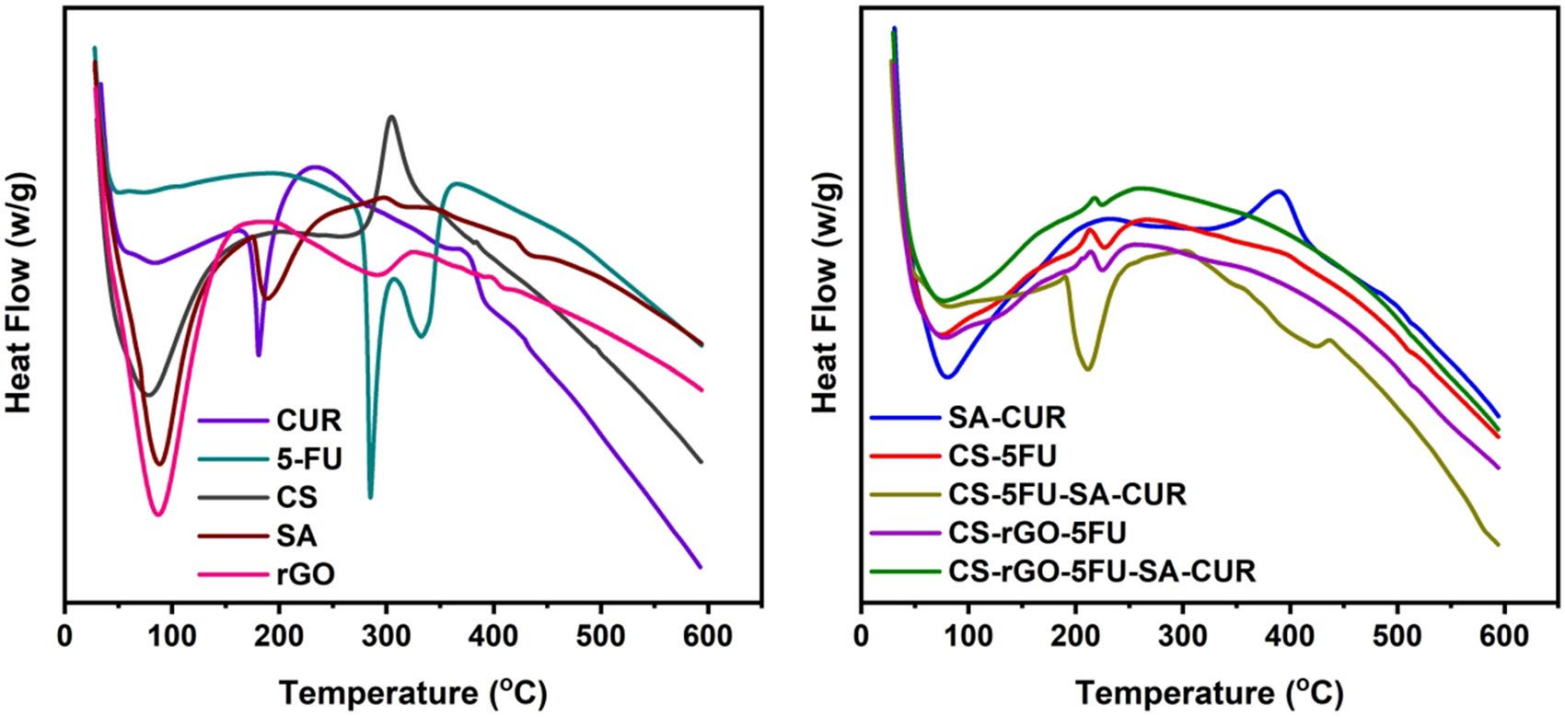
DSC curves of CUR, 5-FU, CS, SA, rGO, SA-CUR, CS-5FU, CS-5FU-SA-CUR, CS-rGO-5FU and CS-rGO-5FU-SA-CUR.

**Figure 5.**
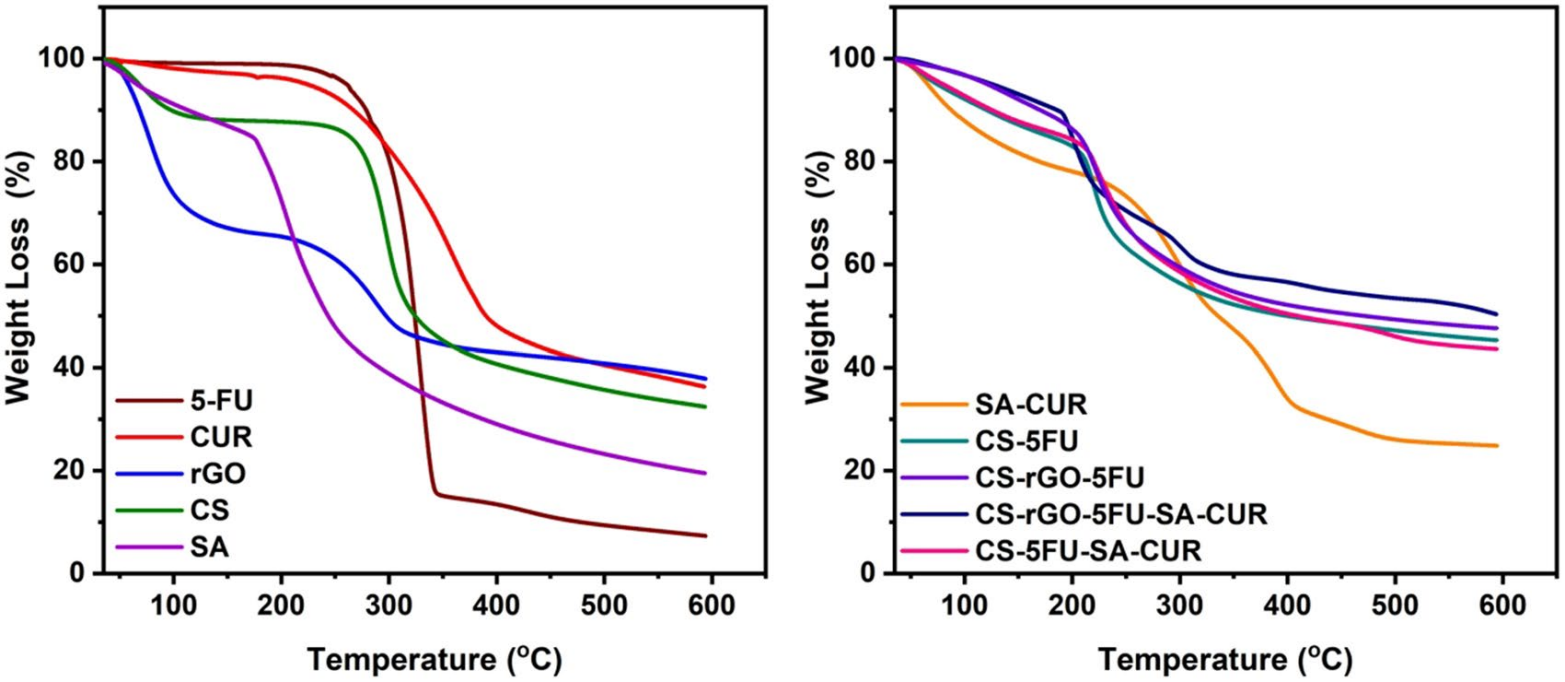
TGA curves of CUR, 5-FU, CS, SA, rGO, SA-CUR, CS-5FU, CS-5FU-SA-CUR, CS-rGO-5FU and CS-rGO-5FU-SA-CUR.

Using thermal analysis (TGA and DSC), the thermal stability of the generated microbeads and the molecular dispersion of drug molecules in the polymer matrix has been investigated. The results are given in Figures 4 and 5. The TGA curve of rGO reveals two distinct weight loss phases. The first phase occurs between 35 °C and 151 °C with a weight loss of 27%. The second occurring between 215 °C and 600 °C with a weight loss of 36%. The first weight loss is caused by the evaporation of adsorbed water, and the second loss is caused by the breakdown of the oxygen-containing functional groups in rGO (Obireddy and Lai 2021a). The TGA curves of CUR and 5-FU are thermally stable up to 169 °C and 195 °C, respectively, after which the compounds begin to degrade and lose weight. At 600 °C, the residual amounts of SA and CS are 19 and 32%, while the residual quantities of SA-CUR and CS-5FU are 25 and 45%, respectively (Giriyappa Thimmaiah et al., 2021). This is because the polymer matrix forms crosslinks with calcium ions, which increases the stability of the polymeric matrix. Similarly, the residual amount of CS-rGO-5FU and CS-rGO-5FU-SA-CUR increased after the inclusion of rGO into microbeads, indicating that the rGO-containing microbeads exhibited excellent thermal stability. CUR and 5-FU’s DSC curves exhibit a sharp peak at 189 °C and 285 °C, demonstrating their respective melting points. These peaks do not show up in microbeads with drugs. This shows that the molecules of CUR and 5-FU are spread out in the polymeric matrix.

### 3.5. Drug loading content and encapsulation efficiency

The EE of all formulations varies depending on the presence of rGO in beads, and the results are listed in Table 1. The incorporation of rGO into the polymeric matrix is the main reason for the higher percentage of EE found in CS-rGO-5FU and CS-rGO-5FU-SA-CUR formulations. The interaction between the functional groups of rGO and the drug moieties provides an explanation for this phenomenon. Because 5-FU forms π-π interactionsinteraction with rGO as well as hydrogen bonds with the –COOH and –OH groups of rGOgroups, the percentage of EE in CS-rGO-5FU and CS-rGO-5FU-SA-CUR formulations increase.

**Table 1.** Encapsulation efficiency of all samples.

Code	DLC (%)		% EE	
	CUR	5-FU	CUR	5-FU
SA-CUR	10.96	NA	51.36	NA
CS-5FU	NA	19.26	NA	71.51
CS-rGO-5FU	NA	20.61	NA	73.26
CUR@CS-5FU-SA-CUR	10.96	NA	50.12	NA
5-FU@CS-5FU-SA-CUR	NA	18.90	NA	69.82
CUR@CS-rGO-5FU-SA-CUR	10.96	NA	50.85	NA
5-FU@CS-rGO-5FU-SA-CUR	NA	20.28	NA	72.48

NA-Not Applicable.

### 3.5. In-vitro release studies and release kinetics

**Figure 6.**
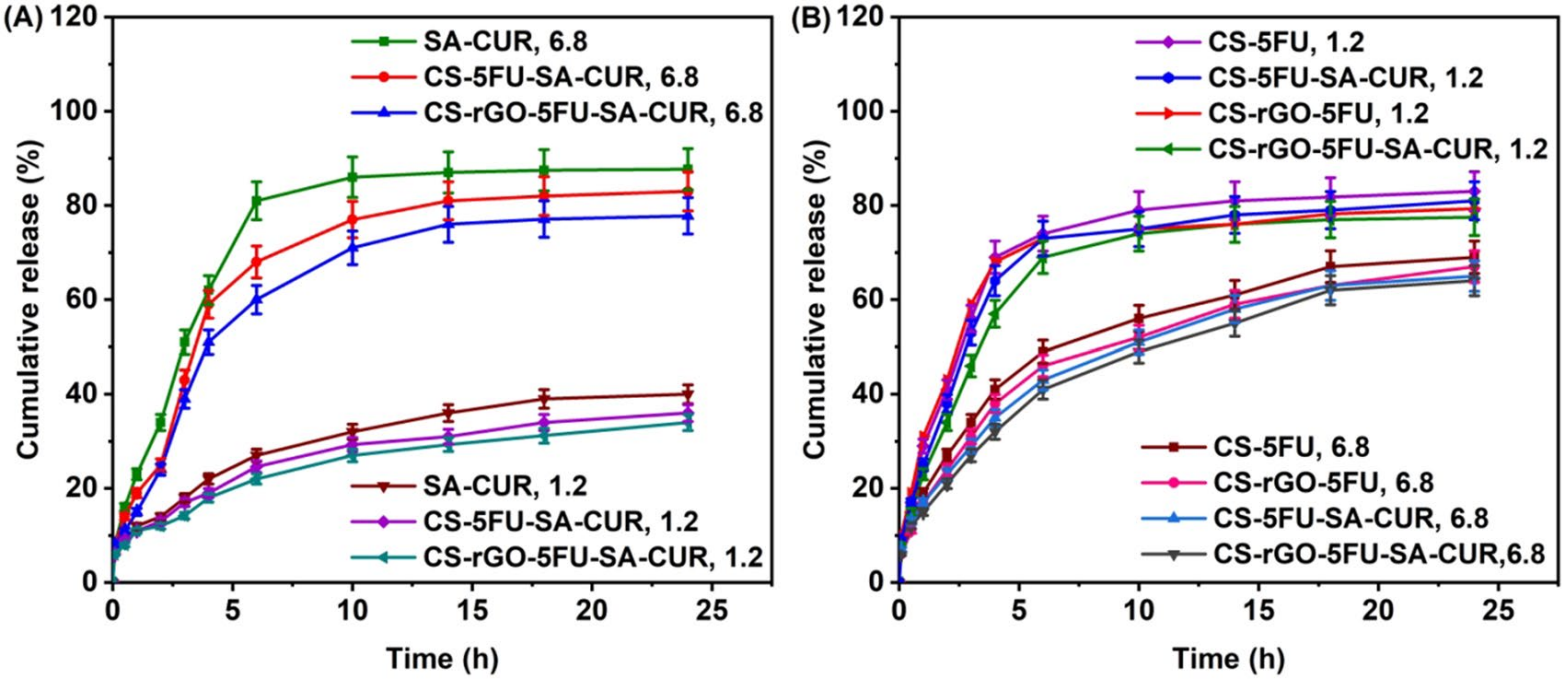
A) CUR release profiles from SA-CUR, CS-5FU-SA-CUR, and CS-rGO-5FU-SA-CUR at pH 1.2 and 6.8; B) A) 5-FU release profiles from CS-5FU, CS-rGO-5FU, CS-5FU-SA-CUR, and CS-rGO-5FU-SA-CUR at pH 1.2 and 6.8.

In vitro release profiles of drug-loaded microbeads have been studied using PBS solution at pH 7.4 and 1.2 at 37 °C, and the findings are shown in Figure 6. From Figure 6, it is interesting to note that the microspheres developed by SA showed good release behavior at pH 6.8 rather than at pH 1.2. This is because the SA matrix interacts less with PBS at higher pH. When this happens, the polymeric matrix becomes loosen, making it easy for the drug molecules to leak out of the network. CS microbeads showed better release behavior at pH 1.2 than at pH 6.8. Because at pH 6.8, chitosan has a lower charge density, the microbeads shrink, whereas, at pH 1.2, there is a possibility that the physical linkages dissociate. The network disintegrates, resulting in a higher release rate. This is in good agreement with the observation made by Zou et al. (2015), who found a similar effect with pH-responsive bovine serum albumin-chitosan microspheres. The drug release was slightly decreases at both pH 1.2 and 6.8 after incorporating rGO into the polymeric matrix. This is because hydrogen bonds are formed between rGO and the polymeric matrix and between rGO and drugs (5FU and CUR), consequently the release rate is slightly lowered at both pH 1.2 and 6.8.

In order to evaluate the mechanism of release kinetics, the correlation coefficient (r^2^) of the linear relationship between the release rate and time has been analyzed for a number of models (zero-order, first order, Higuchi, and Korsmeyer–Peppas), and the results are shown in Table 2. The Korsmeyer–Peppas model is the best appropriate model for understanding the kinetics of drug release from produced carriers, as a correlation coefficient approaching 0.90 is observed. The releasing mechanism is described by the n value in the Korsmeyer-Peppas model. If the n value is 0.43, the drug carriers follow Fickian release. If the n value is 0.85, the drug carriers follow case-II transport. For non-Fickian release or anomalous transport release, the n value lies between 0.43 and 0.85 (Reddy et al., 2019; Chintha et al., 2020). In the present study, the n values of developed carriers are between 0.405 and 0.761 (Table 2), suggesting a non-Fickian release or anomalous transport. The diffusion through the relaxation of polymeric matrix is the key factor in determining drug release.

**Table 2.** Release kinetics parameters of all formulations at pH 6.8 and pH 1.2.

Code	Drug	pH	Korsmeyer-Peppas		Zero Order		First Order		Higuchi	
			n	r^2^	K^0^	r^2^	K_1_	r^2^	K_H_	r^2^
SA-CUR	CUR	6.8	0.715	0.987	5.333	0.213	0.225	0.951	22.83	0.819
		1.2	0.439	0.957	2.244	0.318	0.031	0.540	9.406	0.939
CS-5FU	5-FU	6.8	0.546	0.979	5.068	0.324	0.245	0.848	22.09	0.696
		1.2	0.522	0.996	3.895	0.271	0.083	0.739	16.43	0.925
CS-rGO-5FU	5-FU	6.8	0.576	0.987	4.889	0.230	0.210	0.812	21.22	0.730
		1.2	0.559	0.995	3.689	0.384	0.072	0.762	15.45	0.949
CS-5FU-SA-CUR	CUR	6.8	0.712	0.962	4.929	0.254	0.167	0.915	20.90	0.865
		1.2	0.405	0.934	1.991	0.252	0.026	0.455	8.364	0.923
	5-FU	6.8	0.500	0.978	4.864	0.634	0.244	0.714	21.39	0.584
		1.2	0.496	0.991	3.621	0.401	0.069	0.769	15.14	0.957
CS-rGO-5FU-SA-CUR	CUR	6.8	0.761	0.959	4.567	0.400	0.125	0.902	19.17	0.899
		1.2	0.405	0.939	1.857	0.244	0.031	0.431	7.796	0.925
	5-FU	6.8	0.606	0.994	4.697	0.425	0.168	0.812	20.23	0.795
		1.2	0.526	0.990	3.510	0.506	0.063	0.811	14.57	0.974

### 3.6. In vitro cytotoxicity and ROS generating capacity

**Figure 7.**
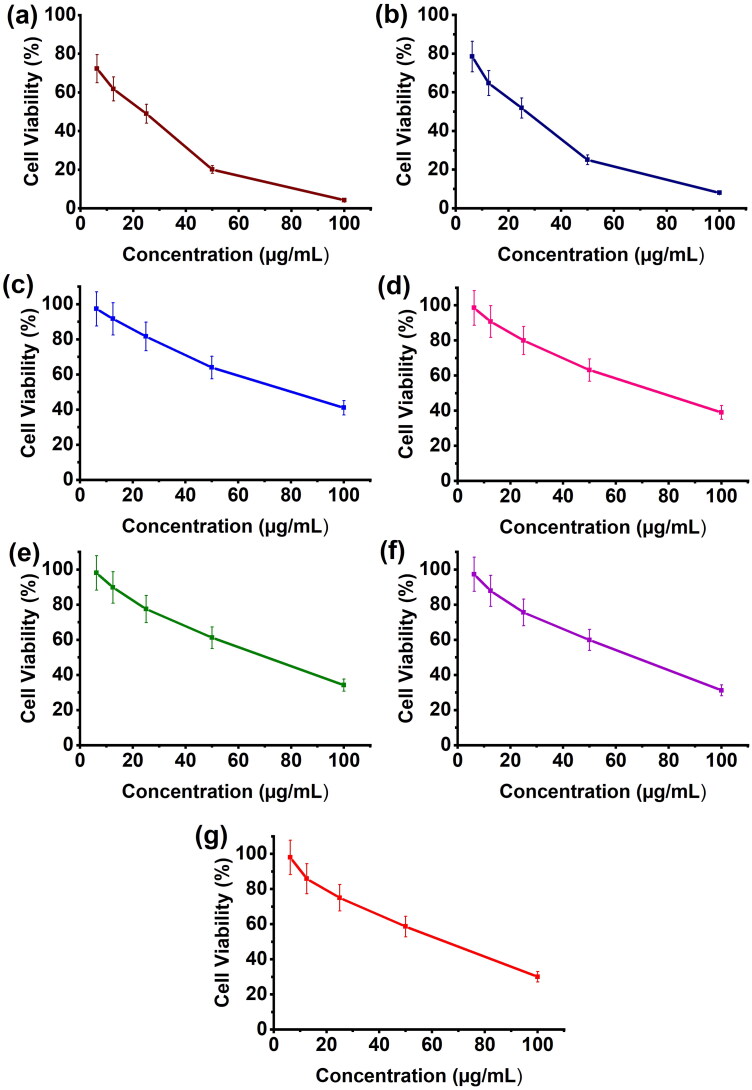
Cell viability of MCF7 cell line against a) 5-FU, b) CUR, c) SA-CUR, d) CS-5FU, e) CS-rGO-5-FU, f) CS-5FU-SA-CUR, and (g) CS-rGO-5FU-SA-CUR.

**Figure 8.**
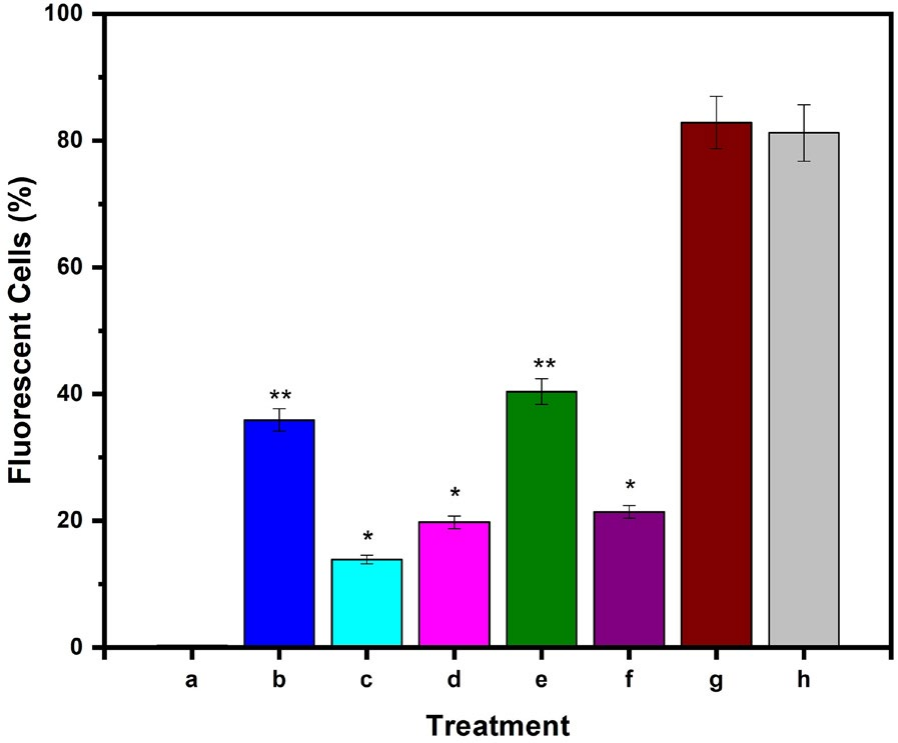
Evaluation of intracellular ROS level in MCF-7 cells up on treatment with a) untreated, b) CS-5FU-SA-CUR, c) SA-CUR, d) CS-5FU, e) CS-rGO-5FU-SA-CUR, f) CS-rGO-5FU, g) CUR and h) 5-FU. Results denote mean ± SD (*n* = 3 per time point). Statistical significance is calculated with respect to free drug by one-way ANOVA (**p < 0.001, *p < 0.01).

The in vitro toxicity of microspheres and microbeads has been studied in MCF7 cells using the MTT assay, and their findings are displayed in Figure 7. The MTT results show that the formulations CS-rGO-5FU-SA-CUR and CS-5FU-SA-CUR have a greater inhibitory effect (% cell viability 31 and 30% respectively) on MCF7 than the other formulations because they contain both drugs and therefore kill more cancer cells. In comparison with CS-5FU (% cell viability 38%), the formulation CS-rGO-5FU (% cell viability 34%) shows good inhibitory performance due to the presence of rGO, which improves the EE of 5-FU and the anticancer property of rGO. This reveals that the generated drug carriers showed good anticancer ability toward MCF-7 cells. Using H2DCFDA labeling, the influence of microbeads on the formation of intracellular ROS has been evaluated. Figure 8 shows that the treatment of the cells increases the amount of endogenous ROS in MCF-7 cells, indicating that the anticancer impact of the drug-loaded carriers is at least to some extent facilitated by ROS production. Previous research has demonstrated that curcumin causes ROS to be produced in a variety of cancer cells as well as apoptosis (Zhang et al., 2015; Araveti & Srivastava, 2019).

## 4. Conclusion

The effectiveness of cancer treatment mediated by combination cancer therapy has been reported to be much higher than that mediated simply by single cancer therapy (Zhou et al., 2013; Butt et al., 2016; Pentak et al., 2016). Over the years, different carriers have been developed (Nicolas & Couvreur, 2017; Piluso et al., 2017; Bolu et al., 2018; Deirram et al., 2019; Khan et al., 2019; Maghrebi et al., 2019), but many of them are designed to deliver only a single agent. Carriers that show multi-drug co-delivery capacity are lacking. This study addresses this problem by developing and characterizing intrinsically bioactive beads showing not only ROS-generating capacity but also pH-responsiveness for multidrug co-delivery in cancer treatment. The beads are produced by using emulsion-templated ionic gelation, which is a simple process imposing little influence on the therapeutic effect of the drugs to be encapsulated inside the beads. Our *in vitro* release studies reveal that the pH responsiveness of the beads provided by CS and SA is different, thereby allowing such responsiveness to be easily tuned in the future by simply controlling the mass percentage of SA and CS in the generated beads. Our beads enable co-delivery of CUR and 5-FU, and have been found to effectively act against MCF7 cancer cells. The effectiveness of our drug-loaded beads to kill cancer cells is further enhanced by the intrinsic ROS-generating capacity exhibited by the beads. All these results show that our beads may work well in combination drug therapy to make cancer treatment more effective.
